# Core needle biopsy as a first-line diagnostic tool for selected thyroid nodules: a real-world evaluation of diagnostic performance and safety

**DOI:** 10.3389/fonc.2026.1707272

**Published:** 2026-03-06

**Authors:** Xing Li, Yi Pan, Yanmei Ou, Xin Gao, Yue Gao, Luwei Liu, Yinze Li, Yong Xu, Wengui Xu

**Affiliations:** 1 Department of Ultrasound Diagnosis and Treatment, Tianjin Cancer Hospital Airport Hospital, National Clinical Research Center for Cancer, Tianjin, China; 2 Department of Ultrasound Diagnosis and Treatment, Tianjin Medical University Cancer Institute and Hospital, National Clinical Research Center for Cancer, Tianjin’s Clinical Research Center for Cancer, Key Laboratory of Cancer Prevention and Therapy, Tianjin, China; 3 Department of Pathology, Tianjin Cancer Hospital Airport Hospital, National Clinical Research Center for Cancer, Tianjin, China; 4 Department of Molecular Imaging and Nuclear Medicine, Tianjin Medical University Cancer Institute and Hospital, National Clinical Research Center for Cancer, Tianjin’s Clinical Research Center for Cancer, Key Laboratory of Cancer Prevention and Therapy, Tianjin, China

**Keywords:** contrast-enhanced ultrasound, core needle biopsy, diagnostic performance, fine-needle aspiration, safety, thyroid nodule, ultrasonography

## Abstract

**Purpose:**

This study aimed to evaluate the diagnostic performance and safety of core needle biopsy (CNB) as a first-line diagnostic tool for selected thyroid nodules with suspicious imaging features or other high-risk characteristics in a real-world setting.

**Methods:**

The protocol for this observational study was approved by the Review Board of Tianjin Medical University Cancer Institute and Hospital. All the medical records of patients who underwent ultrasound (US)-guided CNB of thyroid nodules were searched between 1 January 2022 and 30 April 2023. US-guided CNB was performed using a disposable 18-gauge needle, and the pathological results of CNB were divided into six categories: nondiagnostic, benign, indeterminate, follicular neoplasms (FN) or suspected follicular neoplasms (SFN), suspicion of malignancy, and malignancy. The diagnostic performance and complications of CNB and the risk factors associated with inconclusive results were also assessed.

**Results:**

A total of 286 patients with 316 nodules were included. Of the 199 cases of malignant nodules, 72 were confirmed by surgery after CNB, and 127 were managed as malignant based on definitive CNB findings in conjunction with clinical and imaging correlation, without surgical confirmation. Among the 78 benign nodules, eight were confirmed by surgery, 50 cases were confirmed by CNB plus contrast-enhanced ultrasound (CEUS), and 20 cases were confirmed by CNB with no change in follow-up for more than 1 year. The non-diagnostic and inconclusive rates were 1.9% and 10.4%, respectively. The diagnostic accuracy, sensitivity, specificity, positive predictive value, and negative predictive value of CNB were 98.2%, 100.0%, 93.6%, 97.5%, and 100.0%, respectively. The rate of unnecessary surgeries was 6.3%.

**Conclusion:**

CNB is an effective diagnostic tool for thyroid nodules, demonstrating high diagnostic accuracy and a low rate of non-diagnostic results. It may serve as a viable alternative or complementary first-line diagnostic option for selected nodules, particularly those with suspicious ultrasound features or larger sizes, by providing reliable histological architectural assessment.

## Introduction

Thyroid diseases are highly prevalent, with studies showing that the incidence rate of nodules can reach up to 60% in the general population. Furthermore, the incidence rate of malignant thyroid tumors has doubled in the past 40 years (1, 2) Although the majority of malignant thyroid tumors are papillary carcinomas, which have a favorable prognosis, approximately 10% of papillary carcinomas are already associated with distant metastasis at the time of diagnosis (3). Additionally, other malignant thyroid tumors, such as anaplastic, medullary, and metastatic carcinomas, require early and effective diagnosis to improve the clinical prognosis of patients. For a long time, fine-needle aspiration (FNA) has been considered the preferred and crucial diagnostic tool for evaluating thyroid nodules because of its high cost-effectiveness, simplicity, safety, and low complication rates (4, 5). FNA results are commonly reported using the Bethesda System for Reporting Thyroid Cytopathology, which classifies cytological findings into six diagnostic categories with corresponding malignancy risks and management recommendations. Although this system has been widely adopted, limitations remain in cases requiring architectural assessment. Data have shown that the occurrence rate of indeterminate cytology results ranges from 15% to 42%, while the incidence of non-diagnostic results ranges from 10% to 33.6% (6). According to the guidelines of the American Thyroid Association, it is recommended to repeat FNA for thyroid nodules with non-diagnostic results in the initial FNA. However, the rate of obtaining non-diagnostic results on repeat FNA remains high, reaching up to 50% (7, 8). Furthermore, the probability of encountering atypia of undetermined significance (AUS) or follicular lesion of undetermined significance (FLUS) on FNA ranges from 1% to 27% (9, 10). These lesions are associated with malignancy in varying proportions, ranging from 6% to 30% (11). Therefore, the clinical management of such nodules is controversial, and in some cases, unnecessary diagnostic surgery becomes unavoidable. The exploration of alternative diagnostic tools to overcome the limitations of FNA is, therefore, particularly important. In the 1990s, the core needle biopsy (CNB) technique was first proposed as an alternative to FNA, and in recent years, there has been growing interest in this method (12–14). CNB allows for the acquisition of a larger tissue sample, enabling microscopic evaluation of the cellular and tissue architecture of the lesion, thereby aiding in the diagnostic process. Currently, it is being utilized as a primary diagnostic tool for thyroid nodules in some centers (13–16). However, despite some guidelines stating that CNB is safe, effective, and associated with low complication rates, the supporting evidence is largely based on expert consensus, and there is currently limited research available, resulting in a low level of evidence (17, 18). Therefore, the objective of this study was to investigate the diagnostic performance and complications of CNB in the evaluation of thyroid nodules.

## Materials and methods

### Data collection

The protocol for this observational study was approved by the Review Board of Tianjin Medical University Cancer Institute and Hospital. All the medical records of patients who underwent ultrasound (US)-guided CNB of thyroid nodules were searched between 1 January 2022 and 30 April 2023. None of the patients had previously undergone FNA. The indications for CNB included thyroid nodules with suspicious ultrasound features, defined as Thyroid Imaging Reporting and Data System (TI-RADS) categories 4 or 5, as well as large nodules (typically ≥3 cm) in which histological architecture was considered necessary for an accurate diagnosis.

All patients provided informed consent for CNB and underwent complete blood count, coagulation tests, and hemorrhagic disease screening. The location, size and characteristics of the nodules were recorded using US. Biopsy results and complications after CNB and pathological results of patients who underwent surgery were collected.

For nodules categorized as Category III, IV, or V on CNB, further management decisions were made on an individualized basis through multidisciplinary discussions, incorporating ultrasound risk stratification and patient preference, rather than a predefined protocol mandating repeat CNB or surgery.

### Analysis of US results

**Figure 1:**
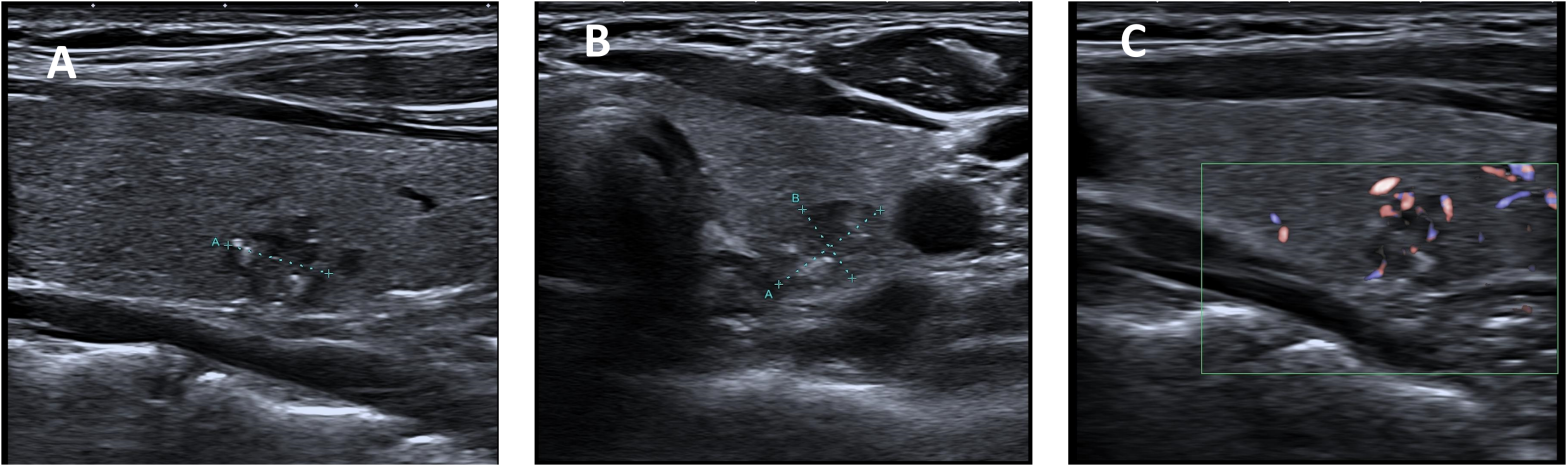
US assessment of thyroid nodule size and ultrasound characteristics. **(A)** Longitudinal view of suspected malignant nodule in the left lobe of thyroid gland **(B)** Transverse view of suspected malignant nodule in the left lobe of thyroid gland **(C)** Color Doppler flow diagram of suspected malignant nodule.

Prior to CNB, all patients’ US examinations were performed by one of the five radiologists under the supervision of the two faculty radiologists. Thyroid nodules were evaluated based on the following features: composition (solid, predominantly solid with some cystic areas, predominantly cystic with some solid areas, or completely cystic), shape (round and oval, taller-than-wide, or irregular), margin (smooth or ill-defined), calcification (microcalcifications, variable-sized calcifications, or coarse calcifications), echogenicity (extremely hypoechoic, hypoechoic, isoechoic, or hyperechoic), and blood flow (minimal or abundant). Solid or predominantly solid composition, taller-than-wide or irregular shape, ill-defined or microcalcifications, or variable-sized calcifications, extremely hypoechoic or hypoechoic echogenicity, and abundant blood flow were all considered indicative features of a potentially malignant nodule (**Figure 1**).

### US-guided CNB technique

One of the following US systems was used for US examinations: Canon (Aplio i700 TUS-AI700, Beijing, China) or Mindray (Diagnostic Ultrasound System, Shenzhen, China). Two radiologists with at least 13 and 25 years of US-guided biopsy experience performed the US-guided CNB procedures.

**Figure 2:**
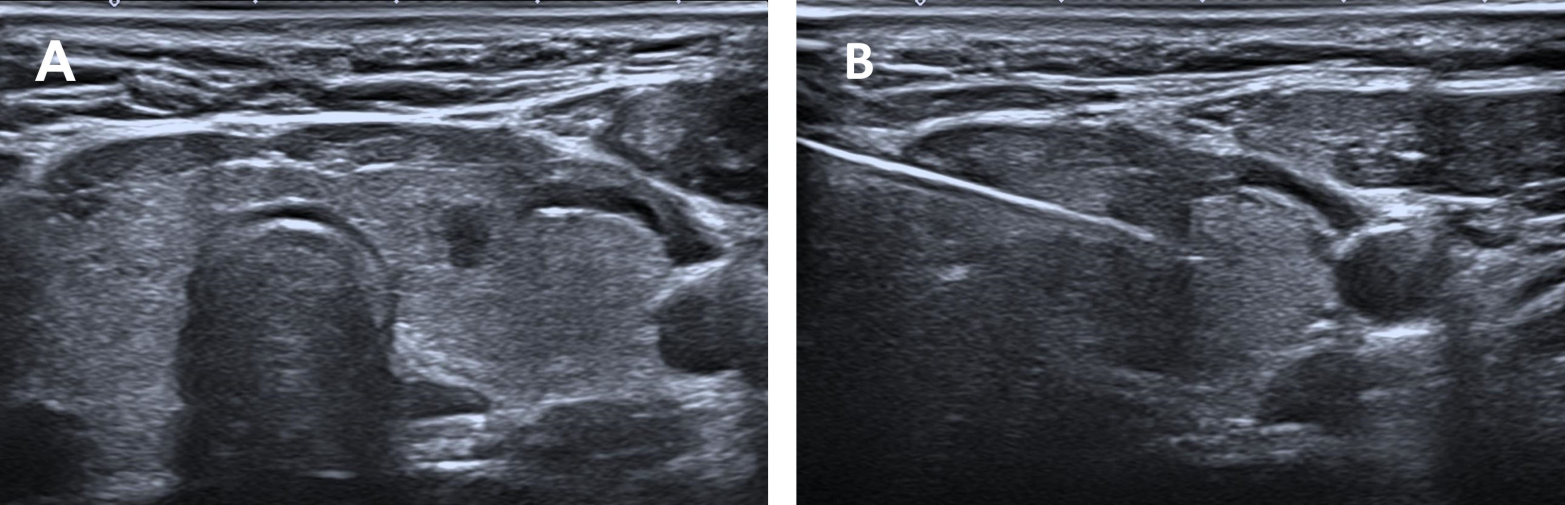
CNB of a small papillary thyroid carcinoma. **(A)** Suspected malignant nodule in the left lobe of the thyroid. **(B)** Biopsy of the lesion with transisthmic access.

**Figure 3:**
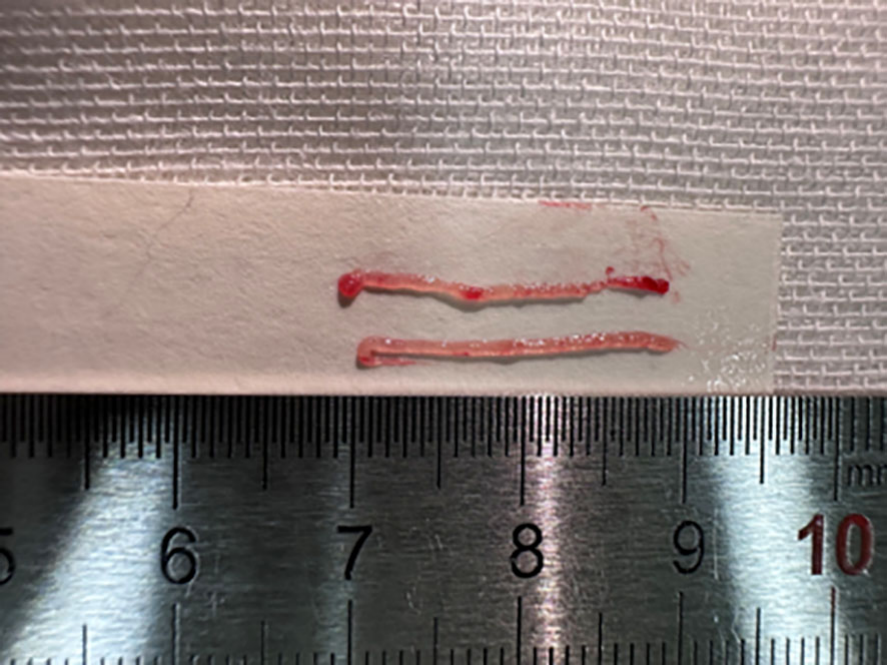
Thyroid nodule specimen by CNB (2cores.).

US-guided CNB was performed using a disposable (1.3 cm, 2.3 cm, or 3.3 cm in length) 18-gauge needle (Argon Medical Devices, Texas, USA). After assessing the nodule using US, local anesthesia with 1% lidocaine was administered at the appropriate location along the access route of the nodule. Using a manual technique, the CNB needle was advanced from the isthmus of the thyroid towards the edge of the thyroid nodule at a distance of 1.3 cm, 2.3 cm, or 3.3 cm (**Figure 2**). Then, a puncture was performed to obtain at least two cores from each nodule. Visual assessment was performed to evaluate the biopsy specimens, including the intranodular tissue and the surrounding normal thyroid tissue (**Figure 3**). The biopsy samples were immediately fixed in 10% formalin solution and sent for further examination. The patient entered an observation period during which local pressure was applied to the puncture site for 10 min–20 min. All patients were routinely observed for approximately 30 min after the procedure. This post-procedural monitoring duration is the standard practice in our institution, primarily to detect immediate complications. Repeat US examinations were performed based on patient complaints.

### Analysis of CNB histopathology

The pathological results of CNB specimens and surgical specimens were reported and reviewed by pathologist with 15 years and 25 years of clinical experience in thyroid pathology, respectively. And all pathological results included immunohistochemistry.

The histologic results of the CNB samples were categorized into six categories according to the proposal approved by the Korean Endocrine Pathology Thyroid Core Needle Biopsy Study Group (19): Category I (nondiagnostic or unsatisfactory) included samples with almost no cells, acellular/low cellularity fibrotic nodules, only normal thyroid tissue, skeletal muscle, mature adipose tissue, and blood clots. If the sample contained atypical cells, regardless of the number of follicular cells present, it should be categorized as requiring diagnostic evaluation. Category II (benign) included all benign thyroid diseases (such as benign follicular nodules, Hashimoto’s thyroiditis, subacute granulomatous thyroiditis, etc.) and non-thyroid lesions (such as parathyroid lesions, benign neurogenic tumors, and benign lymph nodes), or other benign diseases. Category III (indeterminate lesion) included indeterminate nuclear atypia follicular lesions (IIIA) (such as follicular proliferative lesions with focal nuclear atypia, follicular proliferative lesions with ambiguous or suspicious nuclear atypia, and atypical follicular cells embedded in fibrotic stroma), uncertain architectural atypia follicular lesions (IIIB) (such as microfollicular proliferative lesions, solid follicular lesions, and Hürthle cell proliferative lesions lacking fibrous cysts or adjacent normal thyroid tissue, macrofollicular proliferative lesions with a fibrous capsule), and others (IIIC). Category IV (follicular neoplasms or suspected follicular neoplasms) included follicular neoplasms and suspicious follicular neoplasms, where fibrous cysts were present under the microscope, which were different from the adjacent thyroid parenchyma. Category V (suspicion of malignancy) included lesions with histological features that were strongly suggestive of malignancy but insufficient for a definitive diagnosis. In such cases, additional immunohistochemistry can assist with diagnosis (20, 21). Category VI (malignancy) includes lesions exhibiting definite malignant features.

### Analysis of contrast-enhanced US

**Figure 4:**
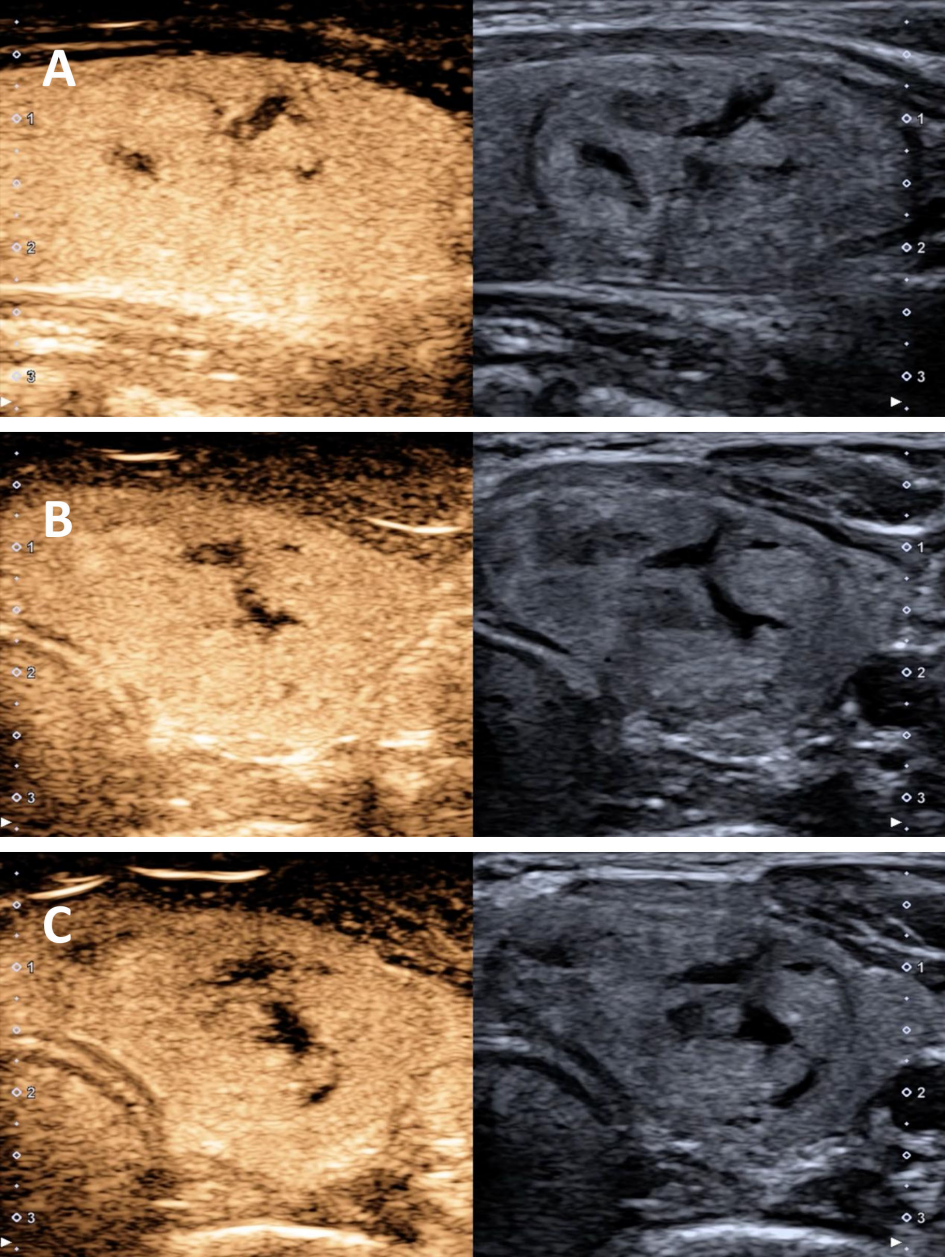
CEUS of benign nodule showed that the arterial and venous stage were enhanced in accordance with the surrounding glands. **(A)** Arterial stage **(B)** Early venous stage **(C)** Late venous stage.

CEUS was used to assist in the diagnosis of nodules with CNB results of Category II. All patients signed an informed consent form before undergoing contrast-enhanced US. The procedure was performed by two radiologists with 13 and 25 years of experience in contrast-enhanced US, respectively. Venous access was established through the elbow vein, and Sonazoid (GE, Healthcare, AS) was used as the contrast agent. Usually, 0.6 ml Sonazoid was used, followed by a flush with 5 ml saline. Continuous dynamic images were recorded for at least 2 min to compare the enhancement differences between the thyroid nodule and the surrounding parenchyma. The degree of enhancement was classified as no enhancement, low enhancement, iso-enhancement, and high enhancement, and the homogeneity and heterogeneity of enhancement within the thyroid nodule were also evaluated (**Figure 4**).

### Diagnostic standard

Malignant lesions:

1. surgical confirmation of malignancy.
2. the CNB results were categorized as Category VI.

Benign lesions:

1. surgical confirmation of benign.
2. benign CNB results (Category II) supported by adjunctive evidence, including CEUS findings suggestive of benignity or stability on follow-up imaging for more than one year.

Undiagnosed lesions: No surgery, secondary CNB, CEUS, or loss of follow-up was performed after a CNB diagnosis of category I, III, IV, or V

Inconclusive lesions on CNB: lesions were categorized as Category I or III

Conclusive lesions on CNB: lesions were categorized as Category II, IV, V, VI

Unnecessary surgery: lesions categorized as Category IV or V on CNB were confirmed as benign

### Statistical analysis

Statistical analyses were performed using the SPSS 21.0 software package. Continuous variables are described as means with standard deviations. Statistical differences between groups were compared using an independent t-test. The chi-square test or Fisher’s exact test was used for categorical variables. The sensitivity, specificity, diagnostic accuracy, positive predictive value, and negative predictive value of CNB were calculated to evaluate its performance. Logistic regression analysis was performed to analyze the risk factors associated with inconclusive CNB outcomes. Given the limited number of inconclusive CNB outcomes, only univariate logistic regression was performed to avoid model overfitting and unstable estimate. Statistical significance was set at *P <*0.05.

## Results

### Diagnostic data

**Figure 5:**
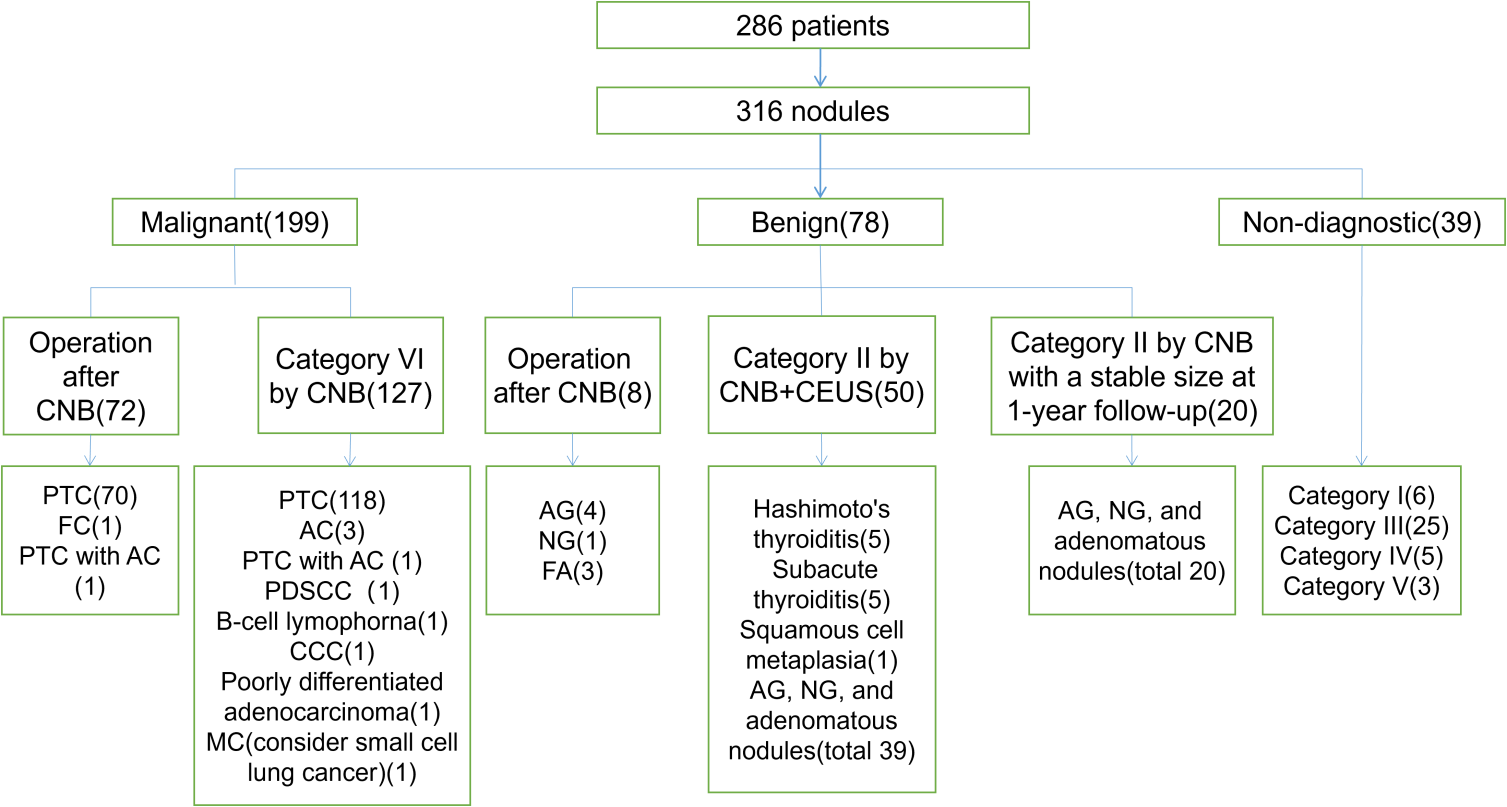
Diagnostic results for patients in this study. The numbers in parentheses represent the number of lesions. PTC, papillary thyroid carcinoma; ATC, anaplastic thyroid carcinoma; FC, follicular carcinoma; PDSCC, poorly differentiated squamous cell carcinoma; CCC, clear cell carcinoma; MC, metastatic carcinoma; AG, adenomatous goiter; NG, nodular goiter; FA, follicular adenoma.

**Figure 6:**
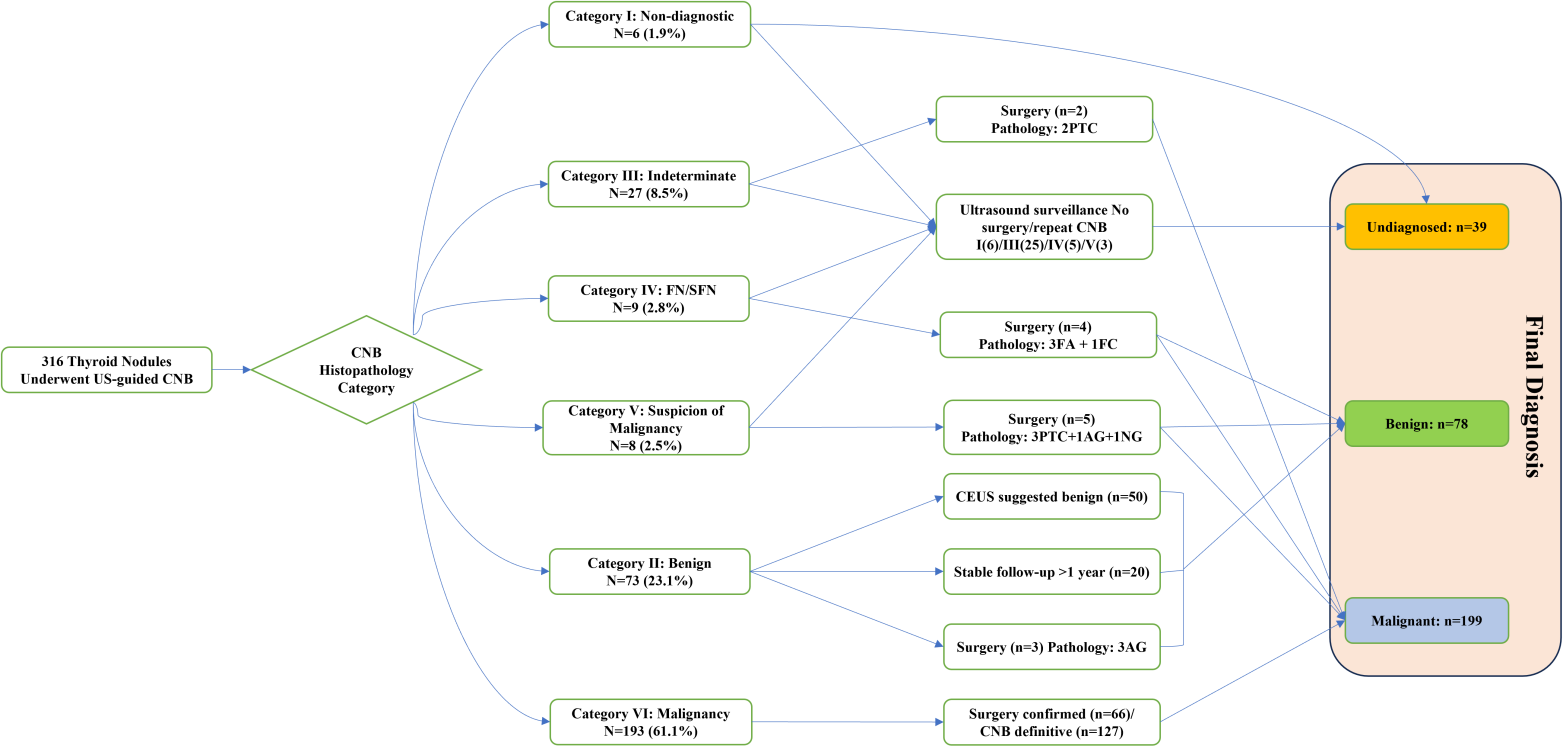
Diagnostic and management flowchart of thyroid nodules using CNB. The numbers in parentheses represent the number of lesions. PTC, papillary thyroid carcinoma; FC, follicular carcinoma; AG, adenomatous goiter; NG, nodular goiter; FA, follicular adenoma; FN, follicular neoplasm; SFN, suspicious follicular neoplasm.

A total of 286 patients with 316 nodules were included in this study. Eighty patients underwent further surgery after CNB, of which 72 had malignant lesions and eight had benign lesions. A total of 193 patients were diagnosed with malignant pathology after CNB (Category VI). For patients with CNB results classified as Category II, CEUS was performed in 50 cases to assist in further confirmation of benign characteristics. Additionally, in 20 cases, the nodules remained stable in size after at least 1 year of follow-up period. There were 39 cases with undiagnosed lesions, including six cases classified as Category I (nodule size ranging from 0.35 cm to 1.0 cm). The CNB results for these cases were as follows: one case showed striated muscle, one case showed striated muscle and fibrofatty tissue, one case showed normal thyroid follicles, two cases showed minimal fibrocalcific tissue, and one case showed cholesterol crystal cleft, multinucleated giant cell reaction, and fibrous tissue proliferation. Twenty-five cases classified as Category III, with the main findings being atypical follicular epithelium and active follicular epithelial proliferation, five cases classified as Category IV, including three cases of follicular tumors and two cases of eosinophilic tumors, three cases classified as Category V, including two highly suspicious papillary carcinomas and one suspicious medullary carcinoma (**Figures 5**, **6**).

### CNB results and final diagnosis of 316 lesions

Of the 316 lesions, 277 had a final diagnosis. Among them, the CNB results of two lesions (Category V) did not match the surgical pathology results: one case had CNB results showing minimally compressed atypical follicular epithelium within fibrous tissue, without excluding PTC, while the surgical specimen confirmed fibrotic nodules with adenomatous thyroid hyperplasia. Another case had CNB results showing predominantly fibrocalcific tissue with very few atypical follicles suspicious for PTC, while the surgical specimen confirmed fibrocalcific nodules with nodular thyroid hyperplasia. Four cases had CNB results (Category IV) suggestive of follicular tumors, which were confirmed by surgical pathology: three cases of follicular adenoma and one case of follicular carcinoma. Two patients had CNB results (Category III) showing atypical follicular epithelium, which was confirmed as papillary carcinoma in the surgical specimen. The specific data of the CNB results (**Table 1**). A proportion of nodules with indeterminate CNB results (Categories III, IV, and V) were managed with ultrasound surveillance rather than immediate repeat biopsy or surgery. During the follow-up period, no aggressive clinical progression was documented.

**Table 1:** CNB results and final diagnosis of 316 lesions.

		Final diagnosis (n = 277)			Final diagnosis (n = 85)			Final diagnosis (n = 192)	
Core biopsy	Total CNB (n = 316)	Benign(n = 78)	Malignant (n = 199)	CNB (≥1 cm) (n = 102)	Benign (n = 45)	Malignant (n = 40)	CNB (<1 cm) (n = 214)	Benign (n = 33)	Malignant (n = 159)
Non-diagnostic or unsatisfactory (I)	6 (1.9)	0 (0)	0 (0)	1 (1.0)	0 (0)	0 (0)	5 (2.3)	0 (0)	0 (0)
Benign (II)	73 (23.1)	73 (93.6)	0 (0)	40 (39.2)	40 (88.9)	0 (0)	33 (15.4)	33 (100.0)	0 (0)
Indeterminate lesion (III)	27 (8.5)	0 (0)	2 (1.0)	11 (10.8)	0 (0)	1 (2.5)	16 (7.5)	0 (0)	1 (0.6)
FN or SFN (IV)	9 (2.8)	3 (3.8)	1 (0.5)	9 (8.8)	3 (6.7)	1 (2.5)	0 (0)	0 (0)	0 (0)
Suspicion of malignancy (V)	8 (2.5)	2 (2.6)	3 (1.5)	4 (3.9)	2 (4.4)	1 (2.5)	4 (1.9)	0 (0)	2 (1.3)
Malignancy (VI)	193 (61.1)	0 (0)	193 (97.0)	37 (36.3)	0 (0)	37 (92.5)	156 (72.9)	0 (0)	156 (98.1)

Partial percentages do not add up to 100% due to rounding, FN, follicular neoplasm; SFN, suspicious for follicular neoplasm.

### Comparison of patient and nodule characteristics of CNB with conclusive and inconclusive results

The results of univariate logistic regression analysis showed that, except for calcification, there was no statistical correlation between the determination of CNB results and the patient’s age, sex, nodule location, and US characteristics. In the inconclusive result group, 18 of 33 nodules (54.5%) exhibited calcifications, which was higher than the 42.8% (121 of 283) observed in the conclusive result group, and the difference was statistically significant (**Table 2**). However, this finding should be interpreted cautiously because of the limited number of inconclusive cases.

**Table 2:** Univariate analysis of risk factors associated with inconclusive CNB results.

Variables	Categories	Conclusive (n = 283)	Inconclusive (n = 33)	*P*
Age, years	43 ± 12.9	43 ± 12.6	46 ± 15.4	0.214
Sex, n (%)	Male	56 (19.8)	9 (27.3)	0.102
	Female	227 (80.2)	24 (72.7)	
Location, n (%)	Left lobe	107 (37.8)	18 (54.5)	0.436
	Right lobe	158 (55.8)	14 (42.4)	
	Isthmus	16 (5.7)	1 (3.0)	
	Posterior of thyroid	2 (0.7)	0 (0.0)	
Nodule size, cm	1.31 ± 1.45	1.33 ± 1.48	1.21 ± 1.15	0.096
	<1.0, n (%)	193 (68.2)	21 (63.6)	0.402
	≥1.0, n (%)	90 (31.8)	12 (36.4)	
Margin, n (%)	Smooth	50 (17.7)	6 (18.2)	0.540
	Ill-defined	233 (82.3)	27 (81.8)	
Composition, n (%)	Solid	267 (94.3)	32 (97.0)	0.985
	Cyst-solid	16 (5.7)	1 (3.0)	
Echogenicity, n (%)	Extremely hypoechoic	98 (34.6)	13 (39.4)	0.306
	Hypoechoic	162 (57.2)	19 (57.6)	
	Isoechoic	23 (8.1)	2 (3.0)	
Shape, n (%)	Ovoid-to-round	112 (39.6)	16 (48.5)	0.398
	Taller-than-wide	166 (58.7)	17 (51.5)	
	Irregular	5 (1.8)	0 (0.0)	
Calcification, n (%)	Microcalcifications	83 (29.3)	7 (21.2)	**0.025**
	Variable-sized calcifications	36 (12.7)	9 (27.3)	
	Coarse calcifications	2 (0.7)	2 (6.1)	
	None	162 (57.2)	15 (45.5)	
Blood, n (%)	Minimal	43 (15.2)	7 (21.2)	0.600
	Abundant	18 (6.4)	2 (6.1)	
	None	222 (78.4)	24 (72.7)	

Partial percentages do not add up to 100% due to rounding. Statistically significant values are identified in boldface.

### Evaluation of diagnostic performance of CNB

In the 316 cases of lesions, the non-diagnostic rate was 1.9%. Based on the results shown in **Figure 5**, the main reasons for this could be inaccurate nodule localization or inadequate follicular cells, rendering the specimens unable to represent US images of the lesions. The conclusive rate was 89.6%, with 33 cases yielding inconclusive results. Apart from the conclusive results, the main category of results was III-class CNB, and patients did not undergo secondary CNB or surgery or were lost to follow-up. The diagnostic accuracy, sensitivity, specificity, positive predictive value, and negative predictive value of CNB were 98.2%,100.0%, 93.6%, 97.5%, and 100.0%, respectively. The unnecessary surgery rate was 6.3% (**Table 3.1**).

**Table 3.1:** The study outcomes of CNB for 316 lessions.*

Study outcomes	Incidence, % (Total)	95% CI
Non-diagnostic or unsatisfactory	1.9 (6 of 316)	0–5.6
Inconclusive	10.4 (33 of 316)	6.6–14.2
Conclusive	89.6 (283 of 316)	86.4–92.8
Malignancy	63.0 (199 of 316)	58.4–67.6
Diagnostic accuracy	98.2 (272 of 277)	95.6–99.3
Sensitivity	100.0 (199 of 199)	97.6–100.0
Specificity	93.6 (73 of 78)	85.0–97.6
Positive predictive value	97.5 (199 of 204)	94.8–100.0
Negative predictive value	100.0 (73 of 73)	93.8–100.0
Unnecessary surgery	6.3 (5 of 80)	0–14.5

CI, confidence interval.

* Diagnostic performance metrics were calculated using a composite reference standard. Results based on surgically confirmed nodules are presented separately in the *Results* section

### Diagnostic performance of CNB in surgically confirmed nodules

Among the 316 nodules, 80 underwent surgical resection and had definitive histopathological confirmation, including 72 malignant and eight benign lesions. To address potential verification bias, the diagnostic performance was recalculated using only surgically confirmed nodules as the reference standard. CNB exhibited a diagnostic accuracy of 93.8%. The sensitivity and specificity were 100% and 37.5%, respectively. The positive and negative predictive values were 93.5% and 100%, respectively. The rate of unnecessary surgery among surgically treated nodules was 6.3% (**Table 3.2**).

**Table 3.2:** Diagnostic performance based on surgically confirmed nodules.

Study outcomes	Incidence, % (Total)	95% CI
Diagnostic accuracy	93.8 (75 of 80)	86.2–97.3
Sensitivity	100.0 (72 of 72)	95.0–100.0
Specificity	37.5 (3 of 8)	13.7–69.4
Positive predictive value	93.5 (72 of 77)	85.8–97.2
Negative predictive value	100.0 (3 of 3)	43.9–100.0
Unnecessary surgery	6.3 (5 of 80)	0–14.5

### Evaluation of diagnostic performance of CNB by subgroup analysis based on nodule size

Subgroup analysis revealed that among the 316 lesions, malignant nodules accounted for a higher proportion of lesions <1 cm (74.3%), significantly higher than that of nodules ≥1 cm (39.2%). The CNB diagnostic method demonstrated a diagnostic accuracy, specificity, and positive predictive value of 100% for nodules <1 cm, which were all higher than those for nodules ≥1 cm, with statistically significant differences (**Table 4**).

**Table 4:** The study outcomes of subgroup analysis according to nodule size.

Study outcomes	Incidence, % (<1 cm)	95% CI	Incidence, % (≥1 cm)	95% CI	*P*
Non-diagnostic or unsatisfactory	2.3 (5 of 214)	0.3–4.4	1.0 (1 of 102)	0.3–1.6	0.668
Inconclusive	9.8 (21 of 214)	6.1–13.5	11.8 (12 of 102)	5.1–18.5	0.596
Conclusive	90.2 (193 of 214)	86.0–94.4	88.2 (90 of 102)	82.2–94.3	0.596
Malignancy	74.3 (159 of 214)	68.7–79.9	39.2 (40 of 102)	29.7–48.7	**<0.001**
Diagnostic accuracy	100.0 (192 of 192)	97.6–100.0	94.1 (80 of 85)	86.2–97.8	**0.001**
Sensitivity	100.0 (159 of 159)	97.1–100.0	100.0 (40 of 40)	89.1–100.0	–
Specificity	100.0 (33 of 33)	87.0–100.0	88.9 (40 of 45)	75.2–95.8	**0.048**
Positive predictive value	100.0 (159 of 159)	97.1–100.0	88.9 (40 of 45)	75.2–95.8	**<0.001**
Negative predictive value	100.0 (33 of 33)	87.0–100.0	100.0 (40 of 40)	89.1–100.0	–
Unnecessary surgery	0 (0 of 48)	0–9.1	15.6 (5 of 32)	5.9–33.5	**0.008**

CI, confidence interval. Statistically significant values are identified in boldface.

Among the six lesions classified as Category I in the CNB results, five were smaller than 1 cm, while the remaining one was equal to 1 cm. Of the malignant lesions, 74.3% were <1 cm, with the majority being papillary carcinomas. Metastatic carcinoma, lymphoma, and anaplastic carcinoma were predominantly larger lesions (≥4 cm), and all obtained definitive pathological results (Category VI) after CNB. For lesions classified as Category II, subacute thyroiditis and Hashimoto’s thyroiditis were mostly concentrated in areas <3 cm, whereas adenomatous goiter and nodular thyroid goiter were mostly concentrated in the ≥3 cm region. There were nine lesions diagnosed as Category IV in the CNB, all of which were ≥2 cm, with the majority concentrated in the ≥4 cm range (**Table 5**).

**Table 5:** The proportion of CNB diagnosis results in different size nodules.

Nodule size	CNB results (incidence, %)						
	I	II	III	IV	V	VI	Total
<1 cm	5 (2.3)	33 (15.3)	16 (7.4)	0 (0)	4 (1.9)	157 (73.0)	215
≥1 cm, <2 cm	1 (2.6)	7 (18.4)	5 (13.2)	0 (0)	3 (7.9)	22 (57.9)	38
≥2 cm, <3 cm	0 (0)	9 (47.4)	4 (21.1)	1 (5.3)	0 (0)	5 (26.3)	19
≥3 cm, <4 cm	0 (0)	9 (56.2)	0 (0)	2 (12.5)	1 (6.2)	4 (25.0)	16
≥4 cm	0 (0)	15 (53.6)	2 (7.1)	6 (21.4)	0 (0)	5 (17.9)	28

### Complications

No complications such as hemorrhage, edema, infection, or needle tract seeding, occurred in any of the patients.

## Discussion

FNA is widely considered an important tool for diagnosing thyroid nodules because of its safety, reliability, and simplicity. However, FNA has certain limitations: the non-diagnostic rate ranges from 10% to 36% (22–24), and the indeterminate rate ranges from 3% to 18% (23–25). More importantly, FNA has a high false-negative rate of 13.6% to 56.6% for diagnosing benign nodules (26), which may mislead clinicians in choosing the subsequent treatment approach for thyroid nodules. In this study, CNB demonstrated a low non-diagnostic rate of 1.9% (95% CI 0–5.6), an inconclusive rate of 10.4% (95% CI 6.6–14.2), and no false-negative cases were identified during follow-up. These findings suggest that CNB may improve diagnostic adequacy and histological characterization in selected thyroid nodules. Consistent with our results, a large meta-analysis including 13,585 lesions reported pooled non-diagnostic and indeterminate rates of 3.5% (95% CI 2.4–5.1) and 13.8% (95% CI 9.1–20.3), respectively, further supporting the diagnostic reliability of CNB (27). Taken together, our real-world data indicate that CNB is an effective diagnostic approach in appropriately selected patients. Rather than replacing FNA, CNB may serve as a valuable complementary or alternative first-line option in clinical scenarios in which improved tissue architecture assessment is required. However, selection bias was an inherent limitation of this study. Because CNB was used as a first-line diagnostic tool only in nodules meeting specific clinical and ultrasound criteria, the study population represented an enriched cohort with a higher prevalence of malignancy than that of the general thyroid nodule population. Therefore, our results may not be directly generalizable to all thyroid nodules detected in routine screening settings. Current guidelines and consensus statements consider CNB as an alternative or complementary diagnostic tool in selected scenarios, such as nodules with suspicious imaging features, large nodules, or cases requiring histological architecture for diagnosis (18, 28, 29). Our institutional practice is consistent with this selective use paradigm.

As the CNB operation technique is an important factor affecting the diagnostic results, in this study, the CNB operation of 316 nodules was performed by two experienced radiologists in our institution to exclude the influence of personal factors on the diagnostic efficiency of CNB. Among all lesions, six cases (1.9%) had CNB diagnostic results classified as Category I, and these nodules had a maximum diameter ranging from 0.35 cm to 1.0 cm, indicating a small overall volume. Among them, four cases exhibited hypoechoic features, whereas two cases showed markedly hypoechoic features. Three cases displayed variable-sized calcifications, and two displayed large calcifications. Previous research has indicated a correlation between hypoechoic features and calcifications (especially large ones) with increased cell density, thereby increasing the occurrence of non-diagnostic results in FNA (30, 31). In our study, the proportion of calcified nodules was higher in the inconclusive group than in the conclusive group. This association may be relevant to the six cases in our study in which CNB resulted in Category I outcomes. Among the Category VI results in CNB, definite diagnoses of metastatic carcinoma, lymphoma, clear cell carcinoma, and anaplastic carcinoma can be obtained through histopathological analysis, and the nodules of the patients in this study were all >4 cm. Current research indicates a high false-negative rate for FNA in large thyroid nodules (32–34). This highlights the advantage of CNB in terms of its high sensitivity for large thyroid nodules, thereby avoiding diagnostic surgery for such patients and enabling timely and effective treatment guidance for clinicians.

An interesting finding of this study was the apparently higher diagnostic accuracy of CNB in nodules <1 cm than in larger nodules. This observation is counterintuitive, as CNB is generally considered more technically challenging in subcentimeter nodules because of the fixed throw length of the biopsy needle and the increased risk of capsular injury. This result should be interpreted cautiously and is most likely due to selection bias. In our practice, CNB was performed in sub-centimeter nodules only when they were highly suspicious on ultrasound and technically accessible, while nodules considered unsafe or unsuitable were not biopsied. Therefore, these cases do not represent the full spectrum of <1 cm thyroid nodules. From a technical perspective, CNB of small nodules was preferentially performed using a short throw length (1.3 cm) under strict real-time ultrasound guidance, with careful control of the needle trajectory. These factors may have contributed to the favorable diagnostic performance observed in this highly selected subgroup of patients.

In this study, CEUS was used as an adjunctive diagnostic tool to assist in the diagnosis of nodules with CNB results of Category II. CEUS allows the assessment of vascular perfusion within thyroid nodules, capturing real-time representations of nodule characteristics (35). This improves diagnostic accuracy while reducing overdiagnosis and overtreatment, thereby achieving optimal patient outcomes. Furthermore, adverse reactions to CEUS contrast agents are extremely rare, ensuring their safety and reliability (36, 37). Previous studies have demonstrated that CEUS provides incremental value in differentiating between benign and malignant thyroid nodules. A meta-analysis by Trimboli et al. showed that the sensitivity, specificity, positive predictive value, and negative predictive value of CEUS in different studies were 85%, 82%, 83%, and 85%, respectively, using histopathology as the reference standard (38). Another study showed that the accuracy, sensitivity, and specificity of TI-RADS combined with CEUS in distinguishing benign and malignant nodules were 94.02%, 94.74%, and 93.33%, respectively (39). These data support the use of CEUS as a complementary modality to reduce false-negative CNB results in benign-appearing nodules, although it cannot replace histopathological confirmation of malignancy.

Although CNB offers advantages in tissue architecture assessment, FNA remains an effective first-line diagnostic tool in several clinical scenarios. FNA is particularly useful for small nodules with low-risk ultrasound features, cystic lesions, and settings where rapid, minimally invasive, and cost-effective evaluations are preferred (28). Therefore, CNB should be considered a complementary diagnostic option rather than a replacement for FNA, with the choice of technique tailored to the nodule characteristics and clinical context.

However, this study has some limitations. First, this study did not include a direct comparison group that underwent FNA. Therefore, our results should not be interpreted as evidence of the superiority of CNB over FNA. Future prospective studies with parallel CNB and FNA arms are warranted to directly compare the diagnostic yield, cost-effectiveness, and patient-reported outcomes. Second, benign diagnoses in a subset of nodules were determined using non-surgical reference standards, including CEUS findings and imaging stability during follow-up, which may have introduced verification bias and potentially overestimated the specificity and negative predictive value. To mitigate this limitation, we performed a subgroup analysis limited to surgically confirmed nodules only. When the diagnostic performance was recalculated using only surgically confirmed nodules as the reference standard, CNB maintained excellent sensitivity and diagnostic accuracy, although the specificity was relatively lower. This finding is largely attributable to the small number of benign nodules that underwent surgery, which resulted in wide confidence intervals and limited precision for specificity and negative predictive value estimates. Larger prospective studies with a higher proportion of benign surgical cases are required to validate these findings. Another important limitation is that a subset of nodules with indeterminate CNB results did not undergo further pathological evaluations. This introduces uncertainty regarding the true malignant potential of these lesions and raises the possibility of missed malignancies. Category III lesions represent a heterogeneous group with variable risks. Therefore, long-term imaging surveillance remains essential for patients who are managed non-operatively. Based on previous research protocols (26, 40), we defined benign thyroid nodules as those with CNB Category II results and no significant changes over one year of follow-up. However, it cannot be ruled out that some nodules grow slowly and may not change within one year. These patients require ongoing observation and follow-up to differentiate between benign and malignant nodules. In addition, CEUS is not universally accepted as a definitive diagnostic standard for confirming benign thyroid nodules, which may introduce bias when used as a composite reference standard. Moreover, this was a single-center, real-world study, and the selection of CNB as a first-line diagnostic approach was influenced by the institutional practice patterns. In particular, the size threshold for defining large nodules was based on institutional experience rather than a universally accepted cutoff and may have varied across centers. Therefore, caution is warranted when generalizing these findings to other clinical settings. Finally, the diagnostic performance was evaluated using a per-protocol analysis, excluding undiagnosed lesions without definitive reference confirmation. While this approach avoids assumptions regarding the true disease status, it may overestimate diagnostic accuracy compared with an intention-to-diagnose analysis. This study had several statistical limitations. First, only univariate logistic regression was performed to explore the factors associated with inconclusive CNB results. Owing to the relatively small number of inconclusive events, multivariate analysis was not feasible without the risk of overfitting. Second, some diagnostic performance estimates, particularly those with values reaching 100%, were based on small denominators, resulting in wide confidence intervals. Therefore, these results should be interpreted with caution.

## Conclusion

In summary, for operators, successful localization of thyroid nodules while ensuring the safety of the puncture is more than half the success of the CNB procedure, as the CNB can sample a large amount of tissue, thus overcoming the limitations of cytology. Therefore, CNB appears to be an effective diagnostic approach and may represent a viable alternative or complementary first-line option for selected thyroid nodules, particularly those with suspicious ultrasound features or when histological architecture is required for diagnosis.

## Data Availability

The raw data supporting the conclusions of this article will be made available by the authors, without undue reservation.
